# Pore mutation N617D in the skeletal muscle DHPR blocks Ca^2+^ influx due to atypical high-affinity Ca^2+^ binding

**DOI:** 10.7554/eLife.63435

**Published:** 2021-06-01

**Authors:** Anamika Dayal, Monica L Fernández-Quintero, Klaus R Liedl, Manfred Grabner

**Affiliations:** 1 Department of Pharmacology, Medical University of InnsbruckInnsbruckAustria; 2 Institute of General, Inorganic and Theoretical Chemistry, University of InnsbruckInnsbruckAustria; Columbia UniversityUnited States; National Institute of Neurological Disorders and Stroke, National Institutes of HealthUnited States

**Keywords:** voltage-gated ca^2+^ channel, non-conducting dhpr, ca^2+^ permeation, ca^2+^ selectivity, pore binding affinity, skeletal muscle excitation-contraction coupling, *nc* dhpr, Mouse

## Abstract

Skeletal muscle excitation-contraction (EC) coupling roots in Ca^2+^-influx-independent inter-channel signaling between the sarcolemmal dihydropyridine receptor (DHPR) and the ryanodine receptor (RyR1) in the sarcoplasmic reticulum. Although DHPR Ca^2+^ influx is irrelevant for EC coupling, its putative role in other muscle-physiological and developmental pathways was recently examined using two distinct genetically engineered mouse models carrying Ca^2+^ non-conducting DHPRs: DHPR(N617D) (Dayal et al., 2017) and DHPR(E1014K) (Lee et al., 2015). Surprisingly, despite complete block of DHPR Ca^2+^-conductance, histological, biochemical, and physiological results obtained from these two models were contradictory. Here, we characterize the permeability and selectivity properties and henceforth the mechanism of Ca^2+^ non-conductance of DHPR(N617). Our results reveal that only mutant DHPR(N617D) with atypical high-affinity Ca^2+^ pore-binding is tight for physiologically relevant monovalent cations like Na^+^ and K^+^. Consequently, we propose a molecular model of cooperativity between two ion selectivity rings formed by negatively charged residues in the DHPR pore region.

## Introduction

Excitation-contraction (EC) coupling in skeletal muscle does not require Ca^2+^ influx through the sarcolemmal L-type voltage-gated Ca^2+^ channel Ca_V_1.1 or dihydropyridine receptor (DHPR), as was convincingly demonstrated in influential studies nearly half a century ago (Armstrong et al., 1972; Schneider and Chandler, 1973). Contrary to substantial Ca^2+^ influx through cardiac as well as invertebrate muscle DHPRs, which is essential for the Ca^2+^-induced Ca^2+^-release (CICR) mechanism in cardiac-type EC coupling (Endo, 1977; Palade and Györke, 1993; Bers, 2002), Ca^2+^ influx-independent EC coupling in vertebrate skeletal muscle acts by depolarization-induced Ca^2+^ release (DICR). In vertebrate skeletal muscle, voltage-dependent conformational change of the skeletal muscle DHPR is transmitted via protein-protein interaction to the Ca^2+^ release channel - ryanodine receptor (RyR1) in the sarcoplasmic reticulum (SR), inducing its rapid opening. The resulting massive increase in cytosolic Ca^2+^ concentration leads to skeletal muscle contraction (Armstrong et al., 1972; Schneider and Chandler, 1973; Rios and Brum, 1987; Lamb, 2000).

Recently, two independently generated genetic mouse models, the EK mouse (Lee et al., 2015) and the *nc*DHPR mouse (Dayal et al., 2017) revisited the DICR dogma by questioning the role of DHPR Ca^2+^ influx ablation on skeletal muscle performance, fatigue, fiber differentiation, metabolism, and eventually EC coupling. Unexpectedly, despite both the EK and *nc*DHPR mouse models abolish DHPR Ca^2+^ influx, the histological, biochemical, and physiological results obtained from these models are incompatible. The DHPR(E1014K) pore mutation in the EK mouse (Lee et al., 2015), besides abolishing Ca^2+^ influx, resulted in reduced SR Ca^2+^ store replenishment during sustained activity, reduced muscle endurance, decreased muscle protein synthesis, decreased muscle fiber size, a shift in fiber-type specification, and an obese phenotype (Georgiou et al., 2015; Lee et al., 2015). Conversely, the *nc*DHPR mouse model carrying the DHPR(N617D) pore mutation displayed no differences compared to wild-type (wt) mice across a broad range of tests (Dayal et al., 2017). This N→D mutation was previously identified in zebrafish to be responsible for the loss of Ca^2+^ conductance through the DHPR isoform specific for the fast (glycolytic/white) skeletal muscle (Schredelseker et al., 2010). Since both the pore mutants, DHPR(E1014K) and DHPR(N617D) preclude Ca^2+^ influx, the striking differences in muscle performance, muscle metabolism, and muscle fiber-type composition between EK and *nc*DHPR mice (Georgiou et al., 2015; Lee et al., 2015; Dayal et al., 2017) are apparently not due to DHPR Ca^2+^ conductance. Instead, the proposed interpretation for the EK mouse was that mutation E1014K alters DHPR selectivity and thus enables permeation of physiologically relevant monovalent cations like Na^+^ or K^+^ (Bannister and Beam, 2011; Beqollari et al., 2018). Nevertheless, permeability and selectivity properties and hence, the mechanism of Ca^2+^ non-conductance of DHPR(N617D) has so far not been investigated thoroughly.

In this study, we demonstrate that the mutant DHPR(N617D) remains Ca^2+^ impermeant even under conditions known to augment L-type Ca^2+^ currents. Our results explicitly show that the DHPR pore mutation N617D leads to an increase in Ca^2+^ pore binding affinity from ~1 µM (characteristic for wt DHPR) to nM range. This more than fourfold enhanced Ca^2+^ binding affinity is sufficient not only to completely block Ca^2+^ conductance through the mutant DHPR(N617D) but also does not allow permeation of monovalent cations like Cs^+^, Li^+^, and Na^+^ under physiological Ca^2+^ concentrations. This pore blocking mechanism due to atypical high-affinity Ca^2+^ binding in mutant DHPR(N617D) strongly contrasts the pore blocking mechanism by low-affinity Ca^2+^ binding in pore mutant DHPR(E1014K). As known from previous studies (Yang et al., 1993; Ellinor et al., 1995; Sather and McCleskey, 2003) any amino acid substitution in the DHPR selectivity filter (EEEE locus) essentially decreases the Ca^2+^ pore binding affinity from µM to mM range, leading to loss of Ca^2+^ selectivity and Ca^2+^ conductance. Based on our recent findings, we propose a molecular model of cooperativity between the divalent cation selectivity (DCS) locus in the outer DHPR pore region (Cens et al., 2007) and the EEEE locus in the central pore (Sather and McCleskey, 2003). With this model, we can convincingly explain the divergent impacts of both DHPR pore mutations, N617D and E1014K, on Ca^2+^ selectivity and Ca^2+^ conductance and consequently provide an explanation for the incongruences in muscle performance and functioning between the two distinct pore-mutant mouse models. Furthermore, this model of Ca^2+^ selectivity and Ca^2+^ conductance helps us in understanding the Ca^2+^ non-conductance mechanism in previously identified (Schredelseker et al., 2010) additional DHPR pore mutations, E→Q and D→K (in the EEEE locus and DCS locus, respectively) that emerged during evolution of other Ca^2+^ non-conducting DHPR isoforms in skeletal muscle of bony fish.

## Results

### DHPR(N617D) is Ca^2+^ impermeant even under current amplifying conditions

**Figure 1.**
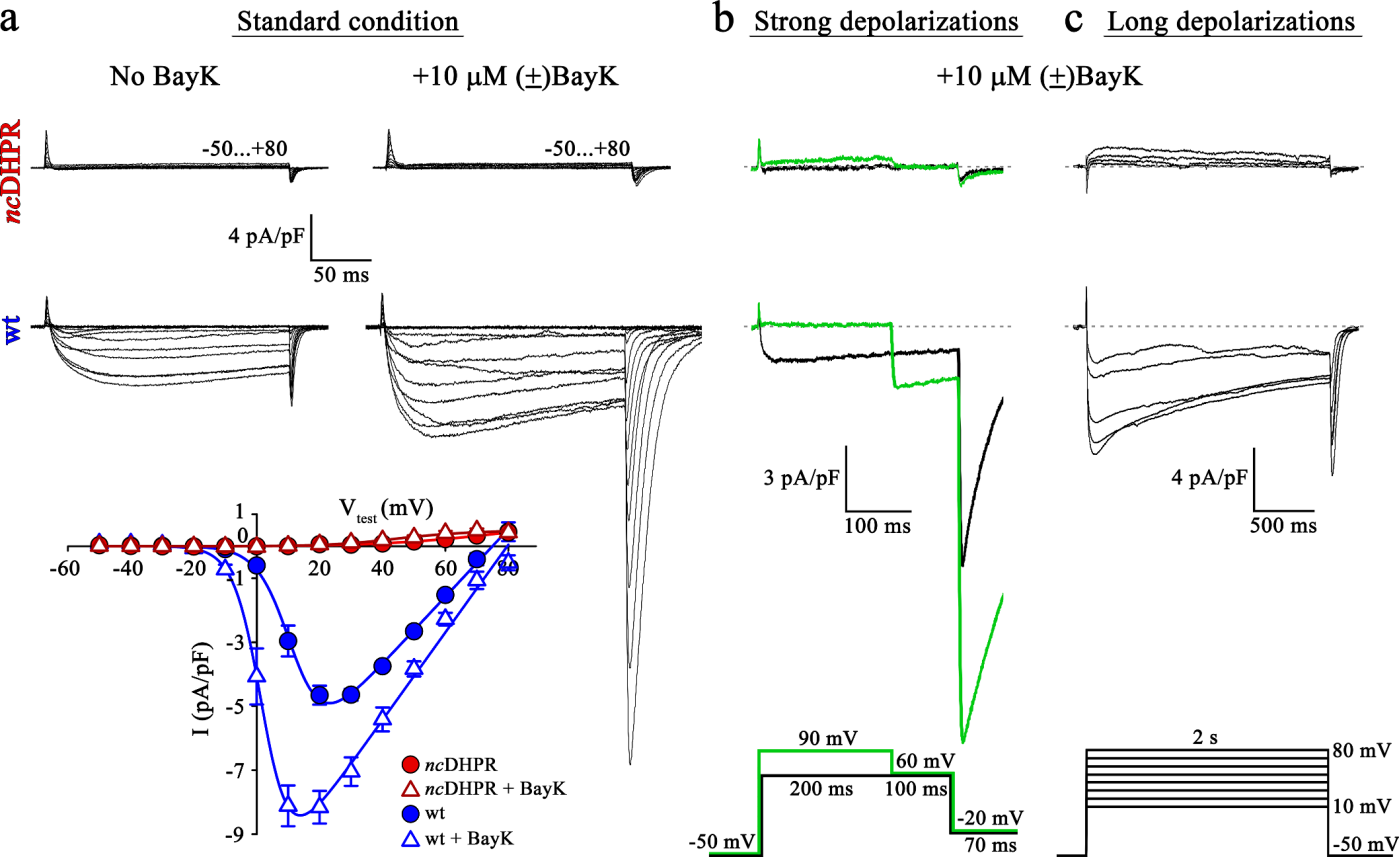
Mutant DHPR(N617D) remains Ca^2+^ impermeant despite strong or long depolarizations in the presence of DHP agonist Bay K. (**a**) Representative whole-cell Ca^2+^ current recordings elicited by 200 ms depolarizations from −50 to +80 mV from *nc*DHPR (*top*) and wt (c*enter*) myotubes before (*left*) and after (*right*) perfusion with 10 µM of the DHP agonist (±)Bay K 8644. Scale bars, 50 ms (horizontal), 4 pA/pF (vertical). Plots of current-voltage relationship (*bottom*) indicates lack of DHPR inward Ca^2+^ currents in the absence (I_max_ = −0.02 ± 0.01 pA/pF; *n* = 8) and presence (I_max_ = −0.02 ± 0.01 pA/pF; *n* = 5) of (±)Bay K through *nc*DHPR myotubes, in contrast to significant (p*<*0.001) augmentation of Ca^2+^ currents in wt myotubes upon administration of (±)Bay K (No Bay K: I_max_ = −5.04 ± 0.27 pA/pF; *n* = 9; with Bay K: I_max_ = −8.82 ± 0.56 pA/pF; *n* = 6). (**b**) 200 ms strong depolarization to +90 mV followed by 100 ms to +60 mV and finally repolarization to −20 mV for 70 ms (*bottom, green lines*) in the presence of 10 µM (±)Bay K, were unable to evoke inward Ca^2+^ currents through DHPR(N617D) (*top*, with +90 mV prepulse: I_max_ = −0.02 ± 0.02 pA/pF; without +90 mV prepulse: I_max_ = 0.01 ± 0.02 pA/pF; *n* = 10). Contrary, wt DHPR displayed significant (p<0.01) depolarization-induced potentiation of inward current at +60 mV (with +90 mV prepulse: I_max_ = −2.97 ± 0.54 pA/pF; without +90 mV prepulse: I_max_ = −1.62 ± 0.37 pA/pF; *n* = 5) (*center*). Upon subsequent repolarization from +60 mV to −20 mV, the tail current was also considerably larger (p<0.01) after the +90 mV pre-conditioning pulse (I_tail_ = −19.36 ± 3.59 pA/pF; *n* = 5) (*center, green trace*) than after the +60 mV pulse (I_tail_ = −10.78 ± 1.99 pA/pF; *n* = 5) (*center, black trace*). Statistical significance was calculated using paired *t*-test. Scale bars, 100 ms (horizontal), 3 pA/pF (vertical). (**c**) Likewise, 2 s long depolarizations from +10 mV to +80 mV in 10 mV increments (*bottom*) in the presence of 10 µM (±)Bay K, were unable to induce Ca^2+^ influx through DHPR(N617D) (*top*, I_max_ = −0.05 ± 0.02 pA/pF; *n* = 5). The same voltage protocol evoked robust inward Ca^2+^ currents through wt DHPR (*center*, I_max_ = −7.69 ± 0.56 pA/pF; *n* = 5). Scale bars, 500 ms (horizontal), 4 pA/pF (vertical). Data are presented as mean ± SEM; p determined by unpaired Student’s *t*-test. Figure 1—source data 1.Data for IV graph.

To investigate whether DHPR pore mutation N617D obstructs Ca^2+^ permeation also under current enhancing conditions, we implemented corresponding experimental protocols and measured whole-cell Ca^2+^ currents from wt and *nc*DHPR myotubes isolated from new born up to 4-day-old mouse pups. As a first step, inward Ca^2+^ currents were recorded in the presence of 10 µM 1,4-dihydropyridine (DHP) agonist (±)Bay K 8644 applied via the standard bath solution (see Material and methods). For voltage-gated L-type Ca^2+^ channels (Ca_V_), Bay K 8644 acts as a channel opener by occupying a fenestration site at the interface of repeats III and IV in the pore region (Grabner et al., 1996; Zhao et al., 2019). Although the standard depolarization protocol (−50 to +80 mV) elicited the expected robust (±)Bay K-induced amplification (p<0.001) of Ca^2+^ currents (No Bay K: I_max_ = −5.04 ± 0.27 pA/pF; *n* = 9 and with Bay K: I_max_ = −8.82 ± 0.56 pA/pF; *n* = 6) through the wt DHPR (Figure 1a, *center* and *bottom*), no inward Ca^2+^ currents (p<0.001) (I_max_ = −0.02 ± 0.01 pA/pF; *n* = 5) or tail currents were evoked in *nc*DHPR myotubes under (±)Bay K 8644 administration (Figure 1a, *top* and *bottom*).

L-type Ca^2+^ channels show a shift in the mode of gating not only by DHP agonist action (Hess et al., 1984) but also in response to strong or prolonged membrane depolarizations. As previously demonstrated (Wilkens et al., 2001), potentiation of L-type Ca^2+^ channels by DHP agonist Bay K 8644 and strong depolarizations occurs via distinct mechanisms. The shift in mode of gating, also referred to as ‘mode 2’ gating is characterized at the single-channel level by high open probability (P_O_) and long mean open times (Pietrobon and Hess, 1990). Depolarization-induced entry into mode 2 is reflected by increased Ca^2+^ currents as well as tail currents with slower rate of current decay. To investigate whether strong depolarizations with simultaneous administration of (±)Bay K 8644 enable the entry of mutant DHPR(N617D) into mode 2 and elicit L-type Ca^2+^ currents, we used the pulse protocol depicted in Figure 1b (*bottom*) (Bannister and Beam, 2011; Bannister and Beam, 2013). Briefly, 200 ms strong, conditioning depolarization pulses from −50 mV to +90 mV, followed by a pulse of +60 mV to putatively elicit enhanced inward Ca^2+^ currents and subsequently a repolarization pulse to −20 mV to trigger tail currents were applied. As expected from wt myotubes, we recorded significantly larger inward Ca^2+^ current at +60 mV (I_max_ = −2.97 ± 0.54 pA/pF; *n* = 5; p<0.01) as well as tail current at −20 mV (I_tail_ = −19.36 ± 3.59 pA/pF; *n* = 5; p<0.01) when preceded by a pulse of +90 mV compared to the corresponding currents recorded without the pre-conditioning depolarization of +90 mV (I_max_ = −1.62 ± 0.37 pA/pF; I_tail_ = −10.78 ± 1.99 pA/pF; *n* = 5) (Figure 1b, *center*). Conversely, no inward currents or tail currents could be evoked in *nc*DHPR myotubes with (I_max_ = −0.02 ± 0.02 pA/pF; *n* = 10) or without the +90 mV pre-conditioning pulse (I_max_ = 0.01 ± 0.02 pA/pF; *n* = 10) (Figure 1b, *top*). The slight outward component at +90 mV is typically observed at strong depolarizing potentials as described previously (Schredelseker et al., 2010; Dayal et al., 2017).

Finally, beside strong depolarizations, long depolarizations are known to drive L-type Ca^2+^ channels into mode 2 state (Pietrobon and Hess, 1990; Bannister and Beam, 2013). Although, the 2 s depolarizations between +10 mV and +80 mV (Figure 1c, *bottom*) in the presence of (±)Bay K 8644 elicited robust, slowly inactivating L-type Ca^2+^ currents in wt control myotubes (I_max_ = −7.69 ± 0.56 pA/pF; *n* = 5) (Figure 1c, *center*), no inward Ca^2+^ currents were evoked in *nc*DHPR myotubes (I_max_ = −0.05 ± 0.02 pA/pF; *n* = 5) (Figure 1c, *top*), suggesting that DHPR(N617D) remained Ca^2+^ impermeant even under potentiating conditions. We found slight outward currents that were similar to previously observed currents in DHPRα_1S_-null (*dysgenic*) myotubes (Bannister and Beam, 2013) recorded under identical conditions, and thus are unrelated to the DHPR.

Altogether, our results demonstrate that recording conditions known to potentiate L-type inward Ca^2+^ currents through the wt DHPR were unable to evoke Ca^2+^ currents through the mutant DHPR(N617D) in the *nc*DHPR mouse model. Out of the three, so far described mutant mammalian DHPR Ca^2+^ channels with ablated Ca^2+^ conducting ability under standard recording conditions, namely R174W (Eltit et al., 2012), E1014K (Lee et al., 2015), and N617D (Dayal et al., 2017), only the voltage-sensor mutant R174W opened partially and produced tail currents under (±)Bay K 8644 administration. This malignant hyperthermia-linked DHPR voltage-sensor mutant R174W also displayed small, but clearly detectable inward Ca^2+^ currents together with enhanced tail currents in response to strong or prolonged depolarizations in the presence of (±)Bay K 8644 (Bannister and Beam, 2013). Integrating previous and present results (Bannister and Beam, 2011), we can conclude that it is impossible to force either of the two DHPR pore mutants, DHPR(N617D) and DHPR(E1014K) into a Ca^2+^ conducting mode by executing the above-described L-type Ca^2+^ current amplifying conditions.

### DHPR(N617D) does not conduct Na^+^ currents

**Figure 2.**
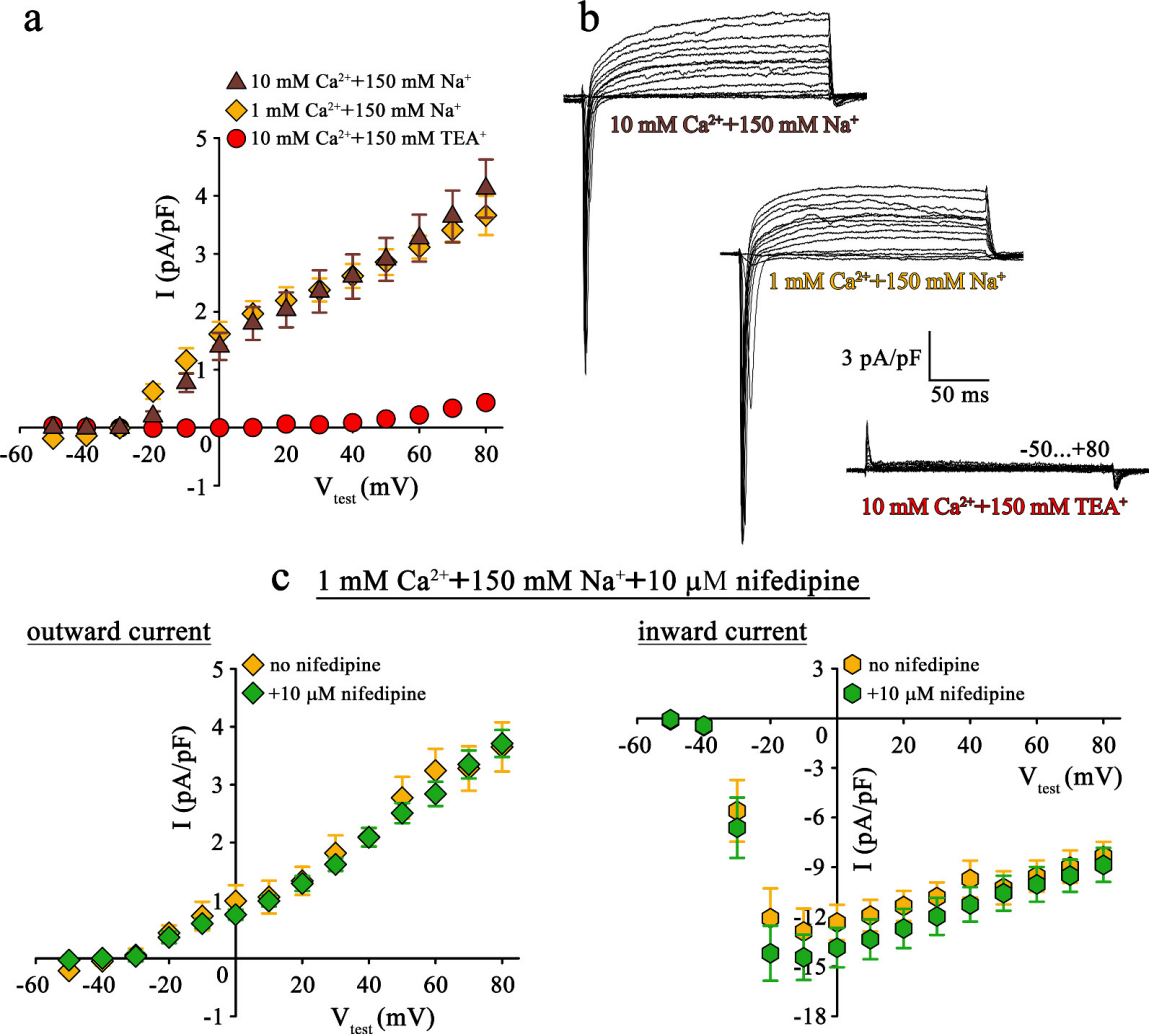
Mutant DHPR(N617D) does not conduct inward Na^+^ currents in the presence of near physiological [Na^+^]. (**a**) Plots of current-voltage relationship for DHPR-mediated Na^+^ currents recorded from *nc*DHPR myotubes indicate the absence of slow-activating, non-inactivating inward Na^+^ currents in the presence of near physiological 150 mM external Na^+^ with either 10 mM (*n* = 8) or 1 mM external Ca^2+^ (*n* = 9). Control recordings were performed in standard bath solution (150 mM TEA^+^, 10 mM Ca^2+^) (*n* = 8). (**b**) Representative current recordings from *nc*DHPR myotubes in response to 200 ms depolarizing test pulses between −50 to +80 mV in the presence of 10 mM Ca^2+^ with either 150 mM Na^+^ (*top*) or 150 mM TEA^+^ (*bottom*), or 1 mM Ca^2+^ with 150 mM Na^+^ (*center*) in the bath solution. Scale bars, 50 ms (horizontal), 3 pA/pF (vertical). (**c**) Plots of current-voltage relationship for *nc*DHPR myotubes at 150 mM external Na^+^ and 1 mM external Ca^2+^ indicate no difference (p*>*0.05) in outward and inward currents in the presence (*n* = 10) and absence (*n* = 6) of 10 µM of the 1,4-DHP antagonist nifedipine. Figure 2—source data 1.Data for IV graphs.

Since both pore mutants, DHPR(N617D) as well as DHPR(E1014K) strictly prevent Ca^2+^ influx even under current enhancing conditions, the striking differences in muscle performance, metabolism, and fiber-type composition between *nc*DHPR and EK mice (Lee et al., 2015; Georgiou et al., 2015; Dayal et al., 2017) can evidently not be due to DHPR Ca^2+^ conductance. However, the reason for these puzzling phenotypic differences could be attributed to distinct selectivity and permeation properties of physiologically relevant monovalent cations through these mutated DHPRs. Basic biophysical characterization of both the DHPR pore mutants, performed either in the respective mouse model (Lee et al., 2015; Dayal et al., 2017) or in heterologous expression systems (Dirksen and Beam, 1999; Schredelseker et al., 2010; Bannister and Beam, 2011; Beqollari et al., 2018) already pointed out substantial differences in monovalent cation conductance. Specifically, under standard Ca^2+^ current recording conditions with 145 mM Cs^+^ present in the patch pipette to block K^+^ channels (Clay and Shlesinger, 1984), massive outward Cs^+^ currents through DHPR(E1014K) (Bannister and Beam, 2011; Lee et al., 2015; Beqollari et al., 2018) but not through DHPR(N617D) (Schredelseker et al., 2010; Dayal et al., 2017; Beqollari et al., 2018) were observed (see also Figure 1a, *top* and *bottom* and Figure 2b, *bottom*).

Apparently, the question arose if this Cs^+^ leakiness of DHPR(E1014K) and tightness of DHPR(N617D) is also factual for other monovalent cations like the physiologically relevant Na^+^ ions. To clarify this conundrum, we performed patch-clamp recordings on *nc*DHPR myotubes under comparable experimental conditions like previously used on DHPR(E1014K) expressed in *dysgenic* myotubes (Bannister and Beam, 2011).

As demonstrated in Figure 2a and b (*top*), at near physiological (150 mM) external Na^+^ as the sole monovalent cation and 10 mM external Ca^2+^, no slow inward Na^+^ currents resembling the L-type Ca^2+^ currents were observed in *nc*DHPR myotubes (*n* = 8). Even upon reducing external Ca^2+^ from 10 to 1 mM to lower the blocking effect by Ca^2+^, DHPR(N617D) remained fully impermeant to Na^+^ ions (Figure 2a and b, *center; n* = 9). Both these observations were in stark contrast to the robust slow-activating, non-inactivating inward Na^+^ currents recorded from DHPR(E1014K) under comparable conditions (Bannister and Beam, 2011). The large, rapidly activating and inactivating inward currents observed within the first ~20 ms after the onset of test potentials can be ascribed to endogenous skeletal muscle Na^+^ channel (Na_V_) isoforms (Numann et al., 1994) and were to a big extent, present even after administration of 2 µM Na^+^ channel blocker tetrodotoxin (Bannister and Beam, 2011). The outward currents observed in the presence of 150 mM external Na^+^ (Figure 2a and b, *top* and *center*) can certainly be ruled out to be Cs^+^ currents through DHPR(N617D) since they are blocked by the K^+^ channel blocker TEA^+^ (Figure 2b, *bottom; n* = 8) and show kinetics very different from Cs^+^ currents recorded from DHPR(E1014K) (Lee et al., 2015; Bannister and Beam, 2011; Beqollari et al., 2018). Moreover, the current-voltage relationship of these currents which start from approximately −30 mV is linear (10 mM Ca^2+^: R^2^ = 0.99; 1 mM Ca^2+^: R^2^ = 0.97) and this together with TEA^+^ sensitivity strongly points to residual K^+^ currents through the endogenous delayed rectifier K^+^ channel (K_V_) (DiFranco and Vergara, 2011). Despite >10 min perfusion with 145 mM Cs^+^ from the patch pipette, these putative K_V_ currents remained unblocked, probably due to limited diffusion in fairly elongated and narrow myotubes.

To directly test for a putative contribution of DHPR(N617D) in mediating the outward and inward currents described above (Figure 2a and b), we measured whole-cell currents in the presence of the 1,4-DHP Ca^2+^ antagonist nifedipine. As depicted in Figure 2c, patch-clamp recordings performed upon addition of 10 µM nifedipine to the bath solution containing 1 mM Ca^2+^ and 150 mM Na^+^, exhibited nifedipine-insensitive slow outward currents (Figure 2c, *left*) and rapidly activating and inactivating inward currents (Figure 2c, *right*). Current-voltage relationship of outward currents (no nifedipine: R^2^ = 0.98; with nifedipine: R^2^ = 0.98) as well as of inward currents (no nifedipine: I_max_ = −13.22 ± 1.41 pA/pF; *n* = 6; with nifedipine: I_max_ = −14.96 ± 1.40 pA/pF; *n* = 10) were unaffected (p>0.05) by the presence of nifedipine. These results unambiguously confirm that DHPR(N617D) is not accountable for the outward and inward currents observed in the presence of near physiological external Na^+^.

### Aberrant high-affinity Ca^2+^ binding to the DHPR(N617D) channel pore

As pointed out above, our results together with previous work (Dirksen and Beam, 1999; Schredelseker et al., 2010; Bannister and Beam, 2011; Lee et al., 2015; Dayal et al., 2017; Beqollari et al., 2018) clearly demonstrate substantial distinct pore properties between DHPR(E1014K) and DHPR(N617D). Although both DHPR pore mutants do not conduct Ca^2+^, DHPR(E1014K) additionally lost its ion-selectivity and robustly conducts monovalent anions like Cs^+^ as well as physiologically relevant Na^+^ and K^+^ even in the presence of physiological concentrations of external Ca^2+^. Although the channel properties of DHPR(E1014K), with its charge conversion of selectivity filter glutamate E_1014_, are accurately explained by a widely accepted model of cardiac Ca^2+^ channel selectivity and permeation (Yang et al., 1993; Ellinor et al., 1995; Sather and McCleskey, 2003) (see Discussion), the non-conductance mechanism of DHPR(N617D) is still unknown (Schredelseker et al., 2010; Dayal et al., 2017).

**Figure 3.**
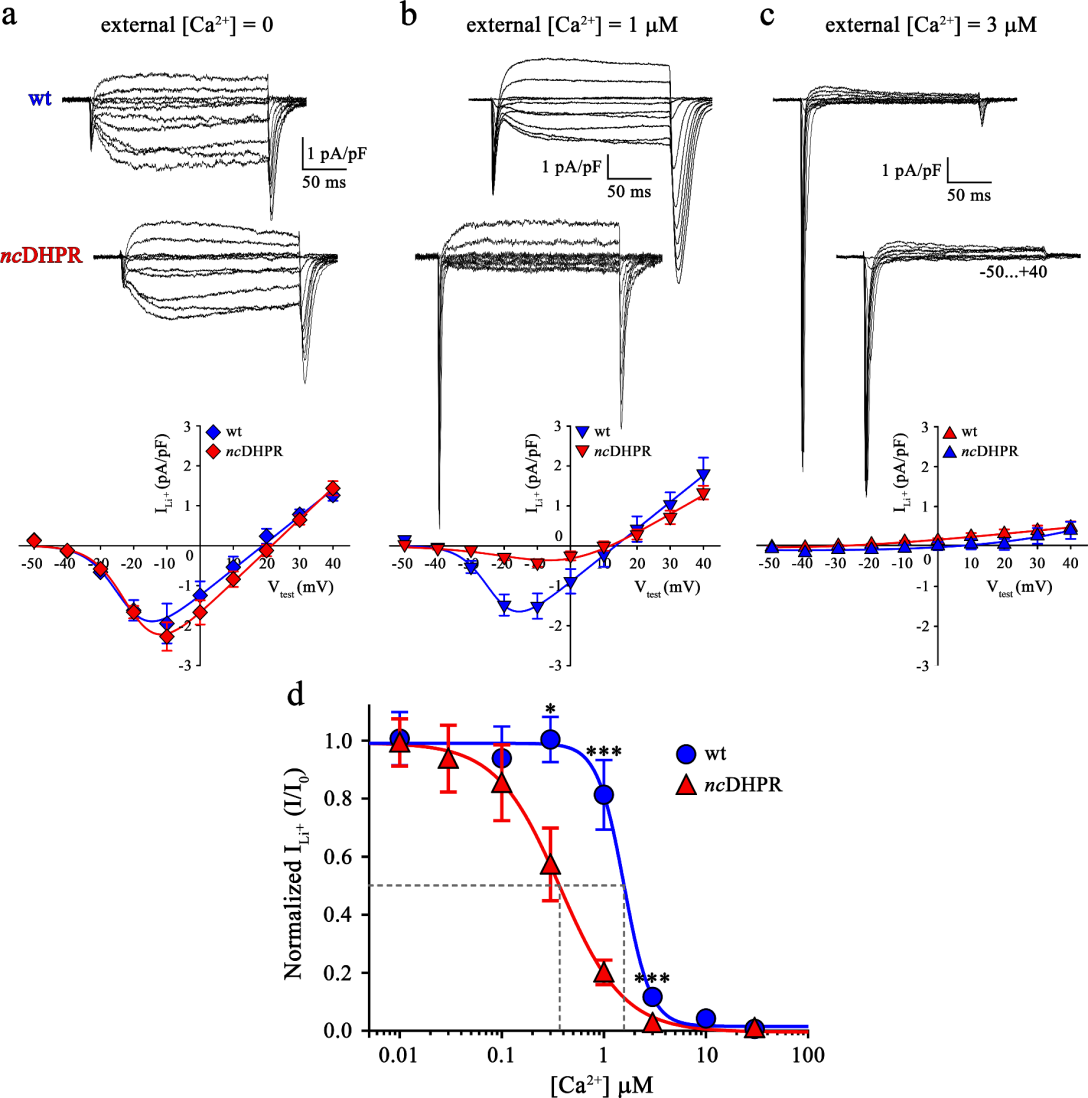
Binding of Ca^2+^ ions with nanomolar affinity within the pore of mutant DHPR(N617D) precludes Ca^2+^ permeation. Representative whole-cell Li^+^ current recordings from wt and *nc*DHPR myotubes in response to 200 ms depolarizations from −50 to +40 mV in the presence of 100 mM external Li^+^ and either 0 (**a**), 1 µM (**b**) or 3 µM (**c**) free external Ca^2+^. Scale bars, 50 ms (horizontal), 1 pA/pF (vertical). Plots of current-voltage relationship are depicted at the bottom of the corresponding representative Li^+^ current traces. Inward Li^+^ currents with no blocking ion (free [Ca^2+^]=0) were indistinguishable (p*>*0.05) between *nc*DHPR (I_max_ = −2.32 ± 0.35 pA/pF; *n* = 16) and wt (I_max_ = −2.07 ± 0.47 pA/pF; *n* = 9) myotubes (**a***, bottom*). However, at higher external [Ca^2+^] of 1 µM (**b**) and 3 µM (**c**), inward Li^+^ currents were significantly (p*<*0.001) smaller in *nc*DHPR (I_max_ = −0.47 ± 0.10 pA/pF, *n* = 8; I_max_ = −0.06 ± 0.02 pA/pF; *n* = 7, respectively) compared to wt myotubes (I_max_ = −1.68 ± 0.25 pA/pF, *n* = 6; I_max_ = −0.24 ± 0.04 pA/pF; *n* = 6, respectively). (**d**) Four-parameter fitted concentration-response curves of Ca^2+^ block of inward Li^+^ currents for wt and mutant *nc*DHPR. Averaged I/I_0_ peak currents are plotted as a function of free external Ca^2+^ concentrations (up to 30 µM) and each data point is an average of 5–16 myotubes (Table 1). There is a significant (p*<*0.01) shift in IC_50_ (*grey dotted lines*) between wt (IC_50_ = 1.57 µM) and *nc*DHPR (IC_50_ = 0.37 µM) indicating a 4.2-fold higher Ca^2+^ pore-binding affinity in the mutant DHPR(N617D) channel. Data are presented as mean ± SEM; p determined by unpaired Student’s *t*-test. Figure 3—source data 1.Data for dose-response graph.

Consequently, we wanted to test if the additional negative charge introduced via the N617D substitution, three residues C-terminal to the selectivity filter glutamate in repeat II and positioned towards the pore entrance (Dayal et al., 2017), enhances Ca^2+^ affinity to the pore and resultantly blocks functional Ca^2+^ permeation by hampering the electrostatic repulsion mechanism (Sather and McCleskey, 2003). As a direct index of Ca^2+^ pore-binding affinity assessment, we performed Ca^2+^ block of Li^+^ current experiments on *nc*DHPR and wt myotubes. In the presence of 100 mM extracellular Li^+^ and without extracellular Ca^2+^ block (free [Ca^2+^]=0), inward Li^+^ currents were indistinguishable (p>0.05) between *nc*DHPR (I_max_ = −2.32 ± 0.35 pA/pF; *n* = 16) and wt (I_max_ = −2.07 ± 0.47 pA/pF; *n* = 9) myotubes (Figure 3a). However, increase in extracellular Ca^2+^ concentration to 1 µM showed a highly significant (p<0.001) reduction of Li^+^ currents through *nc*DHPR (I_max_ = −0.47 ± 0.10 pA/pF; *n* = 8) compared to wt (I_max_ = −1.68 ± 0.25 pA/pF; *n* = 6) myotubes (Figure 3b). These results indicate a higher efficiency of Ca^2+^ block of Li^+^ currents due to enhanced Ca^2+^ binding affinity to the DHPR(N617D) pore. In particular, at 3 µM external Ca^2+^, no Li^+^ currents could be evoked from *nc*DHPR myotubes (I_max_ = −0.06 ± 0.02 pA/pF; *n* = 7) but small, significant (p<0.001) currents through the wt DHPR (I_max_ = −0.24 ± 0.04 pA/pF; *n* = 6) were still existent (Figure 3c). A complete list of peak inward Li^+^ currents at varying free external Ca^2+^ concentrations is presented in Table 1.

**Table 1.** Effect of varying free external Ca^2+^ concentrations on peak inward Li^+^ currents (I_max_) in wt and *nc*DHPR myotubes. I_max_ values of inward I_Li+_ are represented as mean ± SEM with corresponding number of recordings (*n*) from wt and *nc*DHPR myotubes. *p<0.05; ***p<0.001, unpaired Student’s *t*-test.

Free [Ca^2+^]	wt		*nc*DHPR	
	I_max_ (pA/pF)	*n*	I_max_ (pA/pF)	*n*
0	−2.07 ± 0.47	9	−2.32 ± 0.35	16
10 nM	−2.08 ± 0.19	6	−2.30 ± 0.19	12
30 nM	‒	‒	−2.17 ± 0.27	12
100 nM	−1.94 ± 0.23	5	−1.98 ± 0.30	9
300 nM	−2.08 ± 0.16	8	−1.33 ± 0.29 *	8
1 µM	−1.68 ± 0.25	6	−0.47 ± 0.10 ***	8
3 µM	−0.24 ± 0.04	6	−0.06 ± 0.02 ***	7
10 µM	−0.09 ± 0.05	6	‒	‒
30 µM	−0.01 ± 0.02	5	−0.02 ± 0.02	8

**Figure 4.**
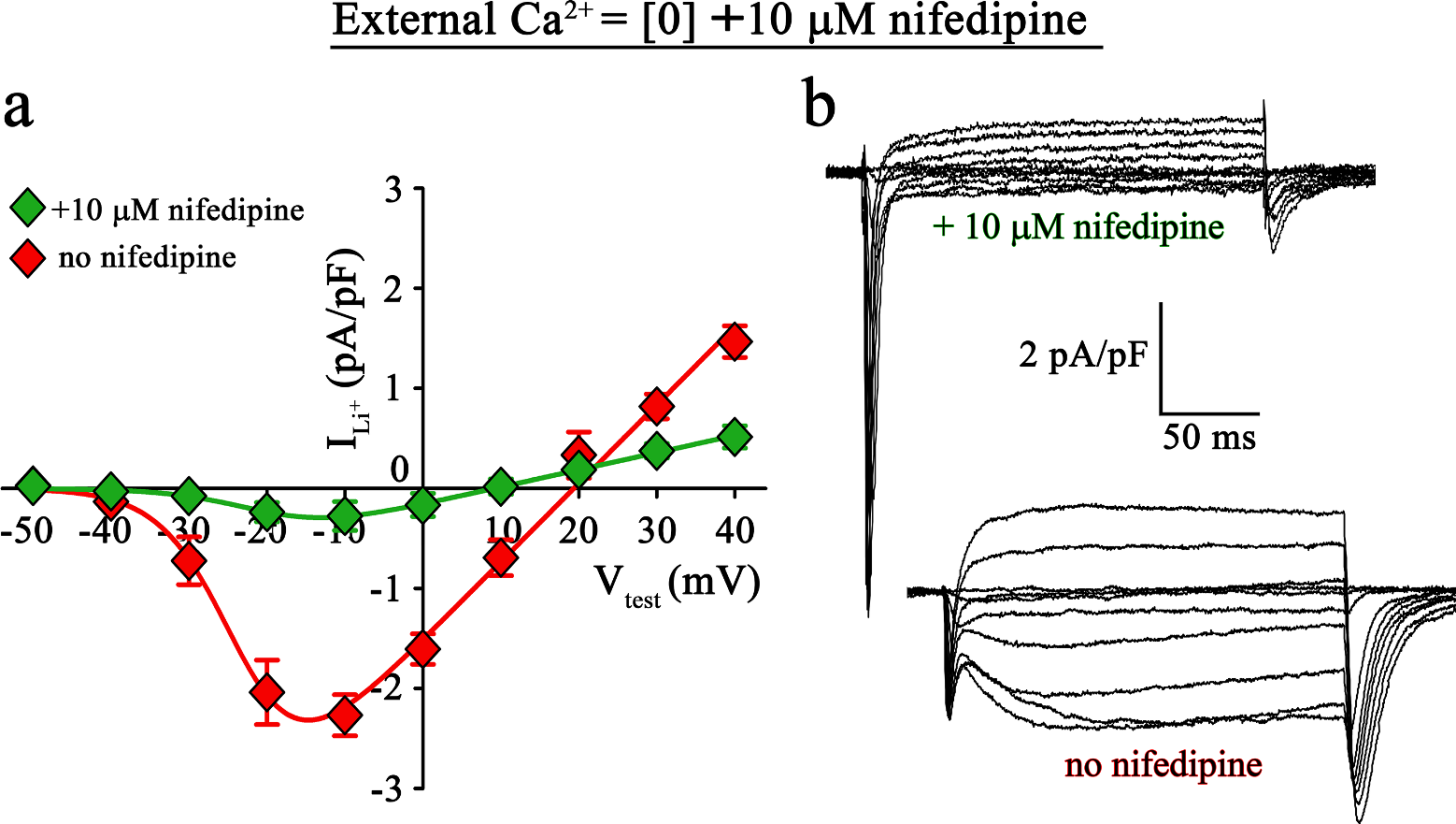
Inward Li^+^ currents conducted by DHPR(N617D) are sensitive to nifedipine block. (**a**) Plots of current-voltage relationship for DHPR-mediated Li^+^ currents recorded from *nc*DHPR myotubes in the presence (I_max_ = −0.35 ± 0.13 pA/pF; *n* = 16) and absence (I_max_ = −2.41 ± 0.27 pA/pF; *n* = 11) of 10 µM of the 1,4-DHP antagonist nifedipine, 100 mM external Li^+^, and free external Ca^2+^ = [0]. Maximum inward Li^+^ currents were significantly (p*<*0.001) reduced in the presence of nifedipine. (**b**) Representative whole-cell Li^+^ current recordings from *nc*DHPR myotubes in response to 200 ms depolarizations from −50 to +40 mV in the presence (*upper*) and absence (*lower*) of 10 µM nifedipine with 100 mM external Li^+^ and 0 external Ca^2+^ concentration. Scale bars, 50 ms (horizontal), 2 pA/pF (vertical). Data are presented as mean ± SEM; p determined by unpaired Student’s *t*-test. Figure 4—source data 1.Data for IV graph.

To directly validate if the slow inward Li^+^ currents are conducted by DHPR(N617D), we recorded Li^+^ currents in *nc*DHPR myotubes in the presence of the 1,4-DHP antagonist nifedipine. As depicted in Figure 4, recordings performed upon addition of 10 µM nifedipine to the bath solution containing 0 Ca^2+^ exhibited a drastic reduction (p*<*0.001) of slow inward Li^+^ currents (no nifedipine: I_max_ = −2.41 ± 0.27 pA/pF; *n* = 11; with nifedipine: I_max_ = −0.35 ± 0.13 pA/pF; *n* = 16). These results confirm that the slow inward Li^+^ currents observed in the absence of external Ca^2+^ are mediated by DHPR(N617D).

Large, rapidly activating and inactivating inward currents detected in both wt and *nc*DHPR myotubes within the first ~20 ms of the onset of test potentials are Li^+^ currents through endogenous skeletal muscle Na^+^ channels, Na_V_ (Numann et al., 1994; DiFranco and Vergara, 2011). Interestingly, their amplitudes appear to correlate negatively to the slow Li^+^ current amplitudes through wt or mutant N617D DHPRs at different external Ca^2+^ concentrations (Figure 3a-c) and were similarly amplified upon nifedipine block of DHPR(N617D) channels at external free [Ca^2+^]=0 (Figure 4b). A possible competition between Ca_V_ and Na_V_ channels for Li^+^ ions, with Ca_V_ taking the priority was not investigated further in the present study.

Eventually, to quantify the impact of the DHPR pore mutation N617D on Ca^2+^ pore binding-affinity in comparison to wt DHPR, Ca^2+^ concentration-response curves displaying inhibition of peak inward Li^+^ currents by Ca^2+^ were analyzed by a nonlinear fit with variable slope (four parameter). As demonstrated in Figure 3d, Ca^2+^ block of inward Li^+^ currents through the skeletal muscle wt DHPR displays an IC_50_ of 1.57 µM (95% CI: 1.36–1.80 µM), which is highly comparable to published values of cardiac DHPR Ca^2+^ pore binding affinity (Yang et al., 1993; Ellinor et al., 1995; Cibulsky and Sather, 2000; Sather and McCleskey, 2003). Interestingly, pore mutant DHPR(N617D) exhibited an IC_50_ of 372.8 nM (95% CI: 334.4–415.8 nM) which is indeed 4.2-fold shifted to lower Ca^2+^ concentrations. Thus, in the mutant DHPR(N617D), introduction of the negatively charged residue D_617_ into the DHPR pore in close vicinity of the selectivity filter EEEE results in a significant (p<0.01) decrease in IC_50_ from µM to nM concentrations. Notably, also the Hill coefficient (*n*_H_) was significantly (p<0.01) different between wt DHPR (−3.32; 95% CI: −4.68 – −2.61) and DHPR(N617D) (−1.39; 95% CI: −1.59 – −1.23) (see Discussion). Since atypical high-affinity binding of Ca^2+^ to the mutant pore is apparently incompatible with Ca^2+^ conductance, this supports the idea of a mechanism by which the mutant DHPR(N617D) pore is occluded.

## Discussion

Our results and earlier findings of Bannister and Beam, 2011 show that in contrast to DHPR(N617D), DHPR(E1014K) functions as a slow-activating, non-inactivating, junctionally-targeted inward Na^+^ channel. Indeed, this difference in intramuscular Na^+^ conductance could be one of the reasons for the different phenotypes observed with the two non-Ca^2+^ conducting DHPR pore-mutant mouse strains, *nc*DHPR and EK. However, of higher physiological relevance than this Na^+^ conductance of DHPR(E1014K), is probably its additional massive 1,4-DHP-sensitive, non-inactivating outward K^+^ conductance, which again is completely absent in the DHPR(N617D) counterpart (Beqollari et al., 2018). K^+^ accumulation is known to play a crucial role in muscle fatigue (Allen et al., 2008) and hence, in the EK mouse strain this mutationally introduced K^+^ efflux from cytoplasm into the transvers (t)-tubular lumen may exacerbate muscle fatigability during periods of enhanced, repetitive activity (Beqollari et al., 2018). Additionally, K^+^ overload in the t-tubule is expected to induce aberrant muscle membrane excitability, which might be the root cause for the muscle histological and metabolic aberrations observed in EK (Georgiou et al., 2015; Lee et al., 2015) but not in *nc*DHPR mice (Dayal et al., 2017).

Even though the results and interpretations of Beqollari et al., 2018 intriguingly suggest K^+^ permeability through DHPR(E1014K) as the basis for the biophysical differences between mutant strains *nc*DHPR and EK, we have to take into consideration the fact that these data were derived from heterologous expression studies in tsA-201cells and not from isolated EK skeletal muscle fibers. Moreover, considering the short duration (~5 ms) of the skeletal muscle action potential (AP) (Sperelakis et al., 2012), slow DHPR activation kinetics (Schrötter et al., 2017), and relatively strong depolarization (+20 mV) required for detectable K^+^ currents (Beqollari et al., 2018), further studies on intact EK fibers are required to fully understand if K^+^ flux through DHPR(E1014K) during an AP or series of APs has important phenotypic implications or not.

Besides the putative influence of the Na^+^ and K^+^ leakiness of DHPR(E1014K) on the EK phenotype, Lee et al., 2015 and Georgiou et al., 2015 presented an alternative hypothesis, which could particularly explain the metabolic aberrations in the EK mouse model. It was proposed that Ca^2+^ permeation and/or high-affinity Ca^2+^ binding to the DHPR is conformationally coupled to the activation of Ca^2+^ / calmodulin-dependent protein kinase type II (CaMKII) and SR store refilling during sustained muscle activity. Consequently, lack of high-affinity Ca^2+^ binding to the DHPR(E1014K) pore causes a decrease in these Ca^2+^-dependent enzyme activities, ensuing alterations in the downstream Ras/Erk/mTORC1 signaling pathways and as a result decreased muscle protein synthesis and the described muscle physiological aberrations (Georgiou et al., 2015; Lee et al., 2015). Although the results derived from *nc*DHPR mice (Dayal et al., 2017) exclude the significance of DHPR Ca^2+^ permeation, they are consistent with a putative crucial role of high-affinity DHPR Ca^2+^ pore binding (like found in wt DHPR or DHPR(N617D)) for accurate CaMKII activation and thus, intact downstream signaling.

Integration of our recent and previous findings (Bannister and Beam, 2011; Beqollari et al., 2018) helped us in addressing the following questions: How to understand the obvious distinct origin of the non-conductance mechanisms of mutants DHPR(E1014K) and DHPR(N617D)? Why is DHPR(E1014K) leaky for monovalent cations, but DHPR(N617D) preserves its high selectivity for Ca^2+^ ions?

### DHPR pore residues responsible for Ca^2+^ selectivity and Ca^2+^ permeation

In an attempt to answer the above questions, we intend to expand a widely accepted molecular model of Ca^2+^ channel selectivity and permeation based on two elegant studies from the Tsien lab (Yang et al., 1993; Ellinor et al., 1995), and comprehensively discoursed in the review of Sather and McCleskey, 2003. According to this model, one Ca^2+^ ion binds to a single high-affinity site formed by all four glutamates (EEEE locus) of the DHPR selectivity filter. This tight embracement of Ca^2+^ in the DHPR pore is a prerequisite for the high selectivity for Ca^2+^ over Na^+^, K^+^, or other monovalent cations. However, to enable rapid passage of Ca^2+^ through the pore, a two-site mechanism that overcomes this tight Ca^2+^ binding is essential. Accordingly, the EEEE locus has been suggested to be physically flexible. Hence, irrespective that all four selectivity filter glutamates are needed to hold a single Ca^2+^ ion with high affinity (K_D_ ~1 µM), their conformation can rapidly rearrange to accommodate a pair of Ca^2+^ ions within the pore, but then both bound with much lower affinity (apparent K_D_ ~14 mM). This intermediate short-lived low-affinity state, together with a Ca^2+^- Ca^2+^ repulsion mechanism occurring in this doubly occupied pore, whereby one of the occupying Ca^2+^ ions is pushed out to the cytosolic side, is the basis for fast Ca^2+^ ion passage through the pore.

Although the Ca^2+^ selectivity filter in form of the conserved EEEE locus within the pore of high threshold voltage-gated Ca^2+^ channels (HVA VGCC) satisfactorily explains divalent/monovalent ion selection, it neither explains the differences in the selectivity for Ca^2+^ among other divalent ions nor the observed distinct conductances through the different HVA VGCC isoforms (Cens et al., 2007). In their interesting study, Cens et al., 2007 via point mutational analyses and molecular modeling identified a ring of non-conserved negatively charged residues located at homologous positions in each of the four repeats of the DHPR pore, which were responsible for the distinct channel profiles. This ring coined as ‘divalent cation selectivity’ (DCS) locus, is present in different constellations in every VGCC and is located towards the outer channel pore region in close vicinity of the selectivity filter EEEE locus. The DCS locus might constitute an additional, low-affinity Ca^2+^-binding site which, together with distinct negative charges closely adjacent to the EEEE locus (Williamson and Sather, 1999), plays a crucial role in defining and directly participating in the generation of different Ca^2+^ conductances in different HVA Ca^2+^ channels (Cens et al., 2007).

### Ca^2+^ non-selectivity and Ca^2+^ non-permeability of the mutant DHPR(E1014K)

As discussed above, proper Ca^2+^ channel permeation and high selectivity are essentially dependent on a single high-affinity Ca^2+^-binding site formed by all four glutamates of the DHPR selectivity filter to assure tight embracement of Ca^2+^. Any substitution in the EEEE locus abolishes/decreases this high (µM) Ca^2+^ pore binding affinity as demonstrated by Ca^2+^ block of Li^+^ current experiments (Yang et al., 1993; Ellinor et al., 1995; Sather and McCleskey, 2003). Specifically, the strongest impact on the binding affinity was produced by exchange of E in repeat III. The EIIIK mutation drastically reduced the pore’s affinity for Ca^2+^ to 1000-fold, as is depicted by an increase in IC_50_ from ~1 µM to ~1 mM for Ca^2+^ block of I_Li+_ (Yang et al., 1993). Although these classical affinity experiments where performed in the cardiac DHPR, the comprehended selectivity/conductance model appears to be congruent with the skeletal muscle DHPR. Accordingly, the large outward Cs^+^ current found in the skeletal muscle EIIIK mutant DHPR(E1014K) (Bannister and Beam, 2011; Lee et al., 2015; Beqollari et al., 2018), which was not blocked even in the presence of 10 mM external Ca^2+^, was consequently interpreted as an indication of very little residual Ca^2+^ binding within the DHPR(E1014K) pore (Dirksen and Beam, 1999; Beqollari et al., 2018). Similarly, a considerable inward Na^+^ current through EK myotubes despite external Ca^2+^ concentration as high as 10 mM (Bannister and Beam, 2011) again indicates a very marginal, low-affinity binding of Ca^2+^ within the DHPR(E1014K) pore. Consequently, low-affinity pore-bound Ca^2+^ is unable to block the flux of any cation in both directions and hence Ca^2+^ selectivity is abolished in the mutant DHPR(E1014K). In addition, since the EEEE locus is mutated to EEKE, attraction of a second Ca^2+^ and subsequent competition for binding valences with the Ca^2+^ ion that is already bound with low affinity to this EEKE locus is impossible. Absence of this intermediate doubly occupied pore and thus, of the Ca^2+^- Ca^2+^ repulsion mechanism as the basis for fast, unidirectional Ca^2+^ ion passage through the pore is sufficient to explain the lack of Ca^2+^ conductance through the mutant DHPR(E1014K).

### High Ca^2+ ^selectivity and Ca^2+ ^non-permeability of the mutant DHPR(N617D)

**Figure 5.**
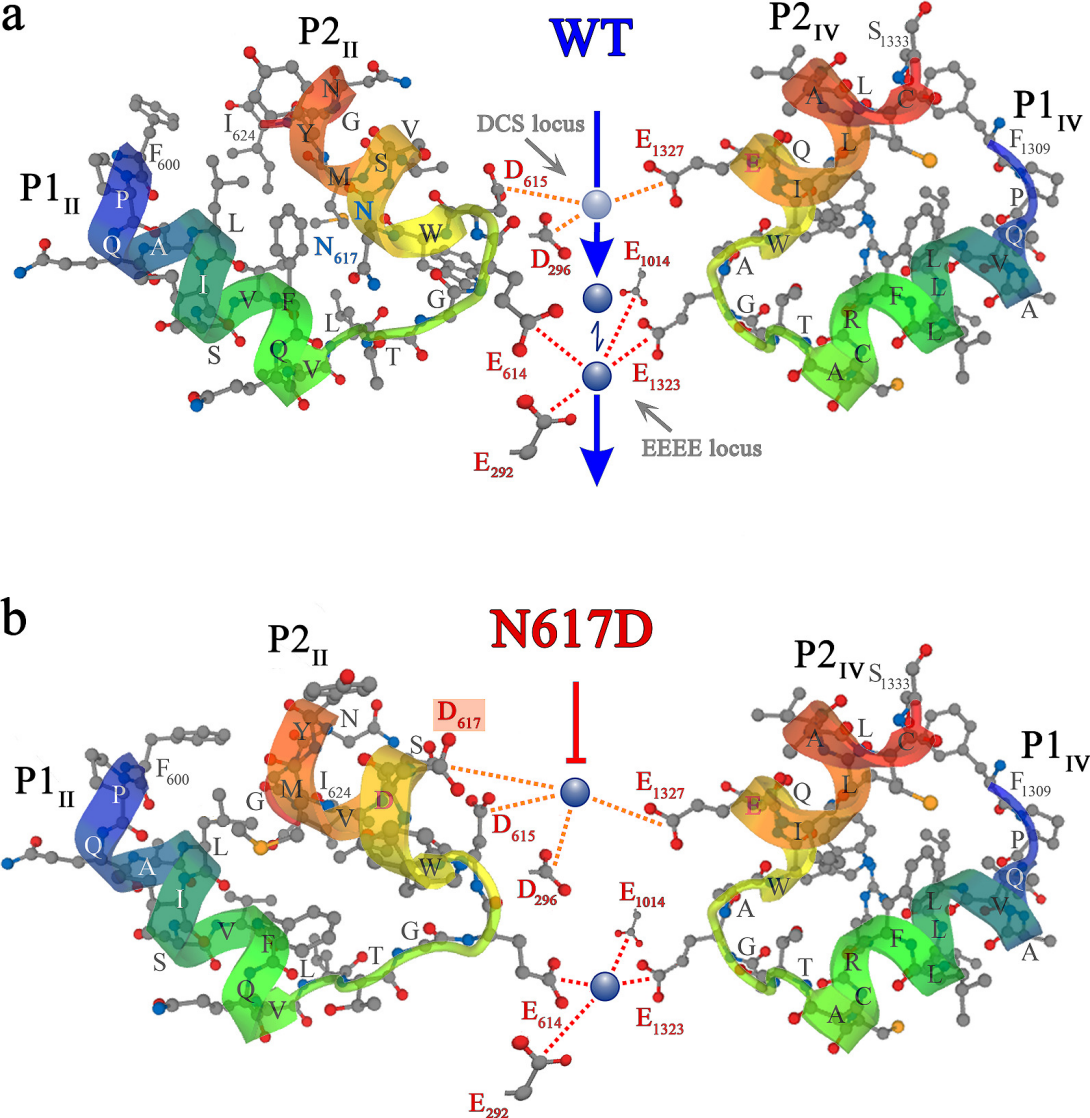
Ca^2+^ selectivity and conductance mechanisms in the wt and mutant DHPR(N617D) channel pore. (**a, b**) De novo conformation prediction of peptide F_600_ - I_624_ constituting the selectivity filter and adjacent pore helices P1 and P2 of DHPRα_1S_ repeat II (P1_II_, P2_II_) (*left*) and of peptide F_1309_ - S_1333_ forming the opposite repeat IV (P1_IV_, P2_IV_) (*right*), using the program PEP-FOLD 3.5 (Thévenet et al., 2012) on the RPBS web portal. Resulting clusters from 200 independent simulations were sorted by sOPEP energy (Wang et al., 2011) to yield the ‘best model’ prediction. Biasing the model prediction of these peptides by imposing the reference structure of DHPRα_1S_ according to the Protein Data Bank (PDB accession number 5GJV) (Wu et al., 2016) did not lead to major differences compared to unbiased modeling approaches and hence we used unbiased models for the wt (**a**) and DHPR(N617D) (**b**) inner channel pore. Depicted best models are graphical overlays of *cartoon* and *balls and sticks* input style options. Models depict the hypothetical mechanism of Ca^2+^ conductance through the wt DHPR (**a**) and the block of Ca^2+^ conductance due to atypical high Ca^2+^ binding affinity (because of introduction of the negative charge D_617_; b*oxed in red*) in the DHPR(N617D) pore region. *Dotted lines* indicate binding interactions between Ca^2+^ ions (*blue spheres*) and carboxyl oxygens (*red balls*) of glutamate E_292_ and aspartate D_296_ of repeat I, E_614_, D_615_, and D_617_ of repeat II, E_1014_ of repeat III, as well as E_1323_ and E_1327_ of repeat IV. Low affinity Ca^2+^ binding is indicated with a *light blue sphere* and high-affinity binding with *dark blue spheres. DCS locus* is the divalent cation selectivity filter (Cens et al., 2007) and *EEEE locus* is the Ca^2+^ selectivity filter. Vertical *blue arrows* indicate active Ca^2+^ conductance pathway in wt DHPR (**a**) and *red T-bar* indicates block of Ca^2+^ flux by aberrant high-affinity binding to the DCS locus in the mutant DHPR(N617D) channel pore (**b**). See Figure 5—figure supplement 1 for additional blocking strategies of DHPR Ca^2+^ conductance in the evolution of skeletal muscle EC coupling.

**Figure 6.**
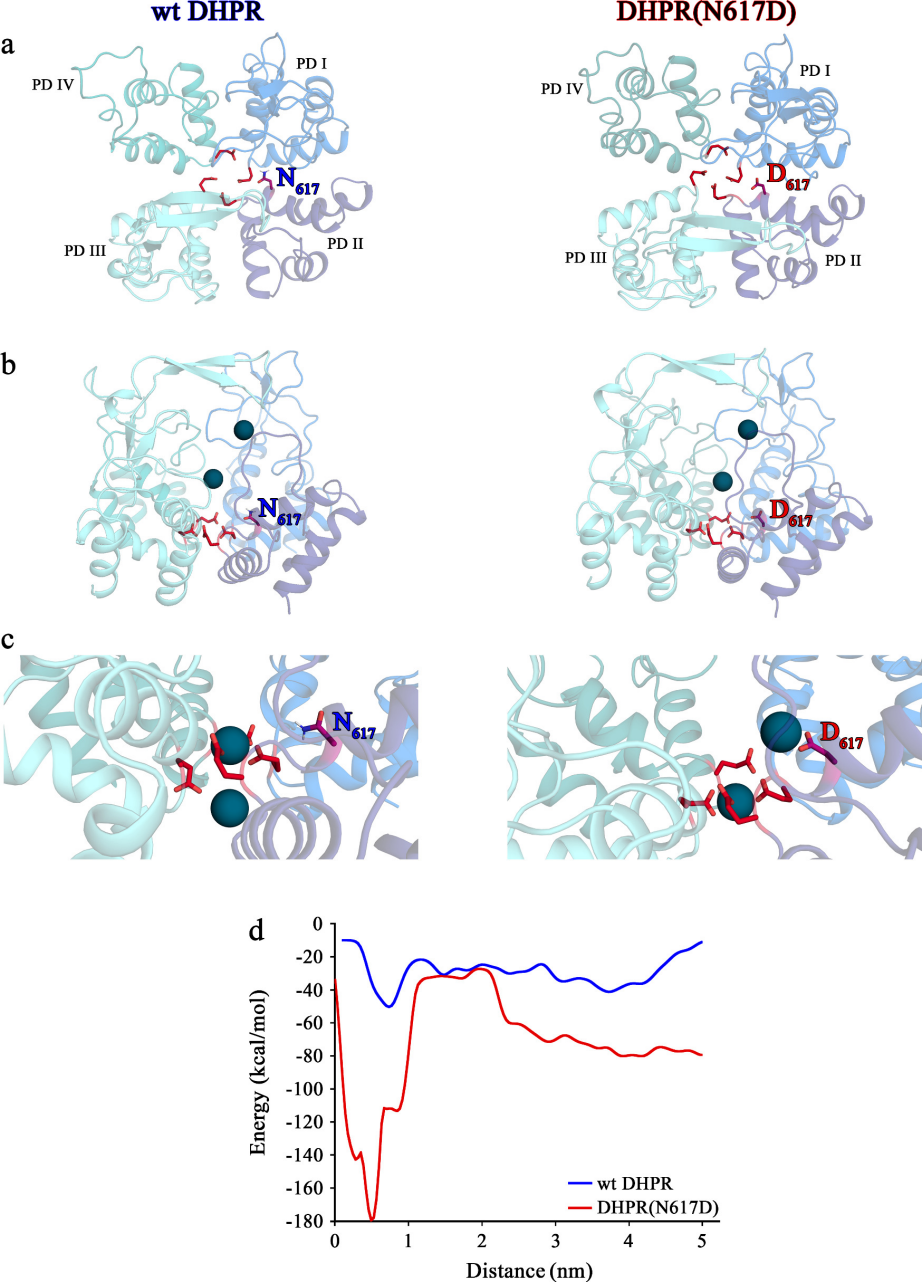
Structure models of selectivity filter regions of wt DHPR (*left panels*) and mutant DHPR(N617D) channel pores (*right panels*) showing the movements of Ca^2+^ ions in simulation studies. (**a**) Top view of the pore illustrating the EEEE and DCS loci. The residues of the EEEE locus are displayed in *red* and the DCS locus is indicated by the position of the residues N_617_ or D_617_. (**b**) Side view of wt DHPR and mutant DHPR(N617D) pores with Ca^2+^ ions present in the pore before starting the equilibration. The *dark blue spheres* represent van der Waals radii of the Ca^2+^ ions. (**c**) Snap-shots immediately following the equilibration run show that Ca^2+^ ions already moved towards the DCS and EEEE loci. While the front Ca^2+^ ion already leaves the selectivity filter of the wt DHPR toward the cytosolic side, Ca^2+^ ions in the DHPR(N617D) pore are still bound to the DCS and EEEE loci. (**d**) Free energy estimations from metadynamics simulations capturing the movements of Ca^2+^ ions through the selectivity filter region. The free energy profile for the passage of Ca^2+^ ions through wt DHPR selectivity filter is depicted in *blue* and for mutant DHPR(N617D) in *red*. The energy barrier of the Ca^2+^ ion leaving the wt DHPR selectivity filter (15 ± 4 kcal/mol; *n* = 5) is significantly smaller (p<0.001) compared to DHPR(N617D) (122 ± 20 kcal/mol; *n* = 5). The process was described by a one-dimensional collective variable that is, the displacement of a Ca^2+^ ion along the axis of the channel pore. A second Ca^2+^ was directly present in the simulation domain. Thus, the energy profile corresponds to the energy experienced by the first Ca^2+^ ion in the presence of the second one. See Figure 6—video 1 and Figure 6—video 2 for illustration of the movement of Ca^2+^ ions through the selectivity filter region of wt DHPR and DHPR(N617D) channel pores, respectively.

Now the question arose, how to understand the pore blocking mechanism observed in DHPR(N617D) by coalescing the models discussed above? Figure 5a depicts the putative mechanism of Ca^2+^ conductance through wt DHPR. The carboxyl oxygens of the DCS locus point toward the pore lumen, allowing coordination of incoming divalent cations with a preference for Ca^2+^ (Cens et al., 2007). According to our postulated pore model (Figure 5a), Ca^2+^ ions from the t-tubular (extracellular) side are attracted to the negative charges of the DCS locus, which in mouse DHPRα_1S_ is formed by D_296_ of repeat I, E_1327_ of repeat IV, and supported by D_615_ of repeat II. This loosely bound Ca^2+^ ion is easily mobilized (probably by charge repulsion from excess Ca^2+^ ions in the t-tubule) and migrates deeper into the pore to compete with the tightly bound Ca^2+^ ion for binding valences of the EEEE locus (in mouse skeletal-muscle DHPR: E_292_, E_614_, E_1014_, E_1323_). Henceforth, due to the reduced binding (µM to mM affinity), Ca^2+^- Ca^2+^ repulsion (Sather and McCleskey, 2003) takes place, eventually pushing the loosely bound Ca^2+^ into the cytosol. This conceptual model is supported by simulation experiments as depicted in Figure 6. Molecular dynamics simulations show that the EEEE locus attracts and stabilizes a single Ca^2+^ ion (Figure 6b–c). However, in the wt DHPR we also observe conformational changes in the EEEE locus that allow binding of a second Ca^2+^ ion. This additional Ca^2+^ ion results in a weaker binding of the glutamate residues to both Ca^2+^ ions, thereby causing a repulsion between the two ions, which is reflected in their decreasing distance to as low as 6 Å (Figure 6c, *left*). Furthermore, metadynamics simulations show that as a consequence of this Ca^2+^ - Ca^2+^ repulsion occurring in the doubly occupied EEEE locus, one of the two Ca^2+^ ions moves toward the cytosolic side (Figure 6c, *left*; Figure 6—video 1). The weaker binding of the Ca^2+^ ions to the EEEE locus of the wt DHPR compared to the mutant DHPR(N617D), is reflected in the significantly (p<0.001) lower free energy barrier (Figure 6d).

**Figure 5—figure supplement 1.**
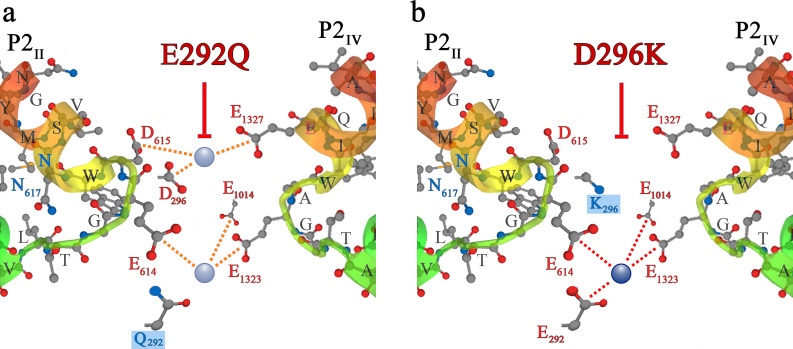
Additional blocking strategies of DHPR Ca^2+^conductance in the evolution of skeletal muscle EC coupling. Symbols and nomenclature are identical to Figure 5. (**a**) Zebrafish slow-muscle specific DHPRα_1S_ carries a distorted EEEE locus, due to substitution of E_292_ of repeat I by Q. Exchange of E_292_ with Q_292_ (*boxed in blue*) in rabbit DHPRα_1S_ yielded mutant DHPR(E292Q) which lacks Ca^2+^ conductance (*red T-bar*) due to alteration of the high-affinity EEEE locus to a low affinity QEEE motif. (**b**) In phylogenetically advanced teleost species, the DCS locus is distorted due to charge conversion, that is exchange of D with K in repeat I. Substitution of D_296_ with K_296_ (*boxed in blue*) in rabbit DHPRα_1S_ yielded mutant DHPR(D296K), which lacks Ca^2+^ conductance (*red T-bar*) due to non-binding of Ca^2+^ to the DCS locus and thus, lack of the attraction mechanism for t-tubular Ca^2+^.

**Figure 6—video 1.** Movement of Ca^2+^ ions through the EEEE locus of the wt DHPR pore toward the cytosolic side. The *dark blue spheres* represent van der Waals radii of Ca^2+^ ions. The residues of the EEEE locus are displayed in *red*.

**Figure 6—video 2.** Movement of Ca^2+^ ions through the DCS and EEEE loci of the DHPR(N617D) pore. Ionic interactions between D_617_ of the DCS locus and the upper Ca^2+^ ion are reflected by the slow detachment and thus slow migration of the Ca^2+^ ion toward the cytosolic side, suggesting occlusion of the pore without the force applied during simulations that pulls the Ca^2+^ ions through the pore.

Contrary to this smooth Ca^2+^- conducting mechanism of wt DHPR, the additional negative charge D_617_ in mutant DHPR(N617D), introduced in the close vicinity to the residue D_615_ in repeat II (Figure 5b), creates an additional binding valence and as a result induces an aberrant high Ca^2+^ binding-affinity to the DCS locus. According to our model, this considerably tighter bound Ca^2+^ is consequently not sufficiently mobile anymore to travel deeper into the pore to compete for the binding valences of the selectivity-filter EEEE locus with the already strongly bound Ca^2+^ ion. Overall, lack of formation of the intermediate short-lived lower-affinity Ca^2+^ binding state, together with the consequential lack of Ca^2+^- Ca^2+^ repulsion at the EEEE locus explicitly explains the absence of Ca^2+^ influx through the DHPR(N617D) pore. Congruently, molecular dynamics simulations show that immediately after the equilibration step, one Ca^2+^ ion is stabilized at the EEEE locus while the other Ca^2+^ is bound to the DCS locus (Figure 6c, *right*; Figure 6—video 2). This translocation of the Ca^2+^ ions to the DCS and EEEE locus occurs already within 1 ns of simulation time succeeding the last step of the equilibration protocol. Here, the distance between the two Ca^2+^ ions is ~9 Å. The strong binding of the two Ca^2+^ ions to the EEEE and DCS locus makes it impossible for any other ion, like Li^+^, to pass through the DHPR(N617D) pore. Thus, simulations of pulling of Ca^2+^ ions through the selectivity filter of mutant DHPR(N617D) result in a significantly (p<0.001), ~8 times higher energy barrier compared to wt DHPR (Figure 6d), which is in accordance with the experimentally observed complete occlusion of the DHPR(N617D) pore in the presence of physiological concentrations of extracellular Ca^2+^ ions (Figure 3). This rather static condition in the DHPR(N617D) pore is well expressed in its lower Hill slope compared to wt DHPR (see Figure 3d). The Hill slope/Hill coefficient (*n*_H_) derived from four parameter logistic fit of dose-response curve is best portrayed as an ‘interaction’ coefficient, reflecting the extent of cooperativity among multiple binding sites (Prinz, 2010). The considerably more dynamic Ca^2+^ interactions in the wt DHPR pore with its successive short-lived intermediate high and low binding affinities and repulsion mechanisms are consequently apparent in the higher *n*_H_ compared to DHPR(N617D).

### Emergence of Ca^2+ ^non-permeant DHPRs during evolution

Point mutation N617D implemented for the creation of mouse model *nc*DHPR (Dayal et al., 2017) was originally identified to be responsible for DHPR Ca^2+^ non-conductivity in zebrafish fast (glycolytic/white) skeletal muscle (Schredelseker et al., 2010). Additionally, with studies on the low-Ca^2+^ conducting DHPR of sterlet (*Acipenser ruthenus*), which is phylogenetically somewhere in between mouse and zebrafish, we showed (Schrötter et al., 2017) that during vertebrate evolution (i.e. from the mammalian species, e.g. mouse, to the teleost fishes, e.g. zebrafish) a steady loss of DHPR Ca^2+^ conductance occurred. Subsuming results of several studies, we proposed the hypothesis that during evolution from mammals to teleost fishes an accumulation of DHPR amino acid exchanges occurred that contributed to the reduction of Ca^2+^ conductance (Schredelseker et al., 2010; Dayal et al., 2017; Schrötter et al., 2017). Mutation N→D (N617D; mouse numbering) that finally ‘turned off’ the already reduced Ca^2+^ conductance evolved only in quite a late phylogenetic stage (Dayal et al., 2017; Schrötter et al., 2017), following the teleost-specific third round (Ts3R) of gene duplication (Meyer and Van de Peer, 2005; Glasauer and Neuhauss, 2014). Beside DHPR non-conductivity, the evolutionary pressure that caused additional substantial modifications in skeletal muscle organization and physiology in teleost fishes (Schredelseker et al., 2010; Dayal et al., 2017; Schrötter et al., 2017) arose from the critical demand for tighter controlled, faster and stronger muscle contractions, crucial for high-speed movements in the aquatic prey-predator environment (Dayal et al., 2019).

Interestingly, Ts3R headed into the evolution of a second DHPR isoform in zebrafish slow (oxidative/red) skeletal muscle that is likewise Ca^2+^ non-conducting (Schredelseker et al., 2010). This slow muscle DHPR is so far the only described innate DHPR with a distorted EEEE locus, where glutamate of repeat I is substituted by glutamine. Exchange of this selectivity filter E_292_ with Q in a GFP-tagged rabbit DHPRα_1S_ clone (Grabner et al., 1998) yielded mutant DHPR(E292Q), which upon heterologous expression in *dysgenic* myotubes confirmed the abolishment of inward Ca^2+^ currents (Schredelseker et al., 2010) with a slight outward Cs^+^ current, typically starting at +20 to+30 mV (Bannister and Beam, 2011). As described earlier (Yang et al., 1993), the EQ pore mutation in repeat I of the cardiac DHPR exerted a minor effect, as the increase in IC_50_ was only twofold compared to the wt. If we assume that a similar right-shift of affinity also holds true for the skeletal muscle DHPR(E292Q), then appropriate Ca^2+^ pore-affinity essential for proper Ca^2+^ selectivity and Ca^2+^ conductance must exist in a surprisingly small range. Incorporation of our present and previously published data (Yang et al., 1993; Schredelseker et al., 2010) indicates that this small range might be within approximately one order of magnitude, somewhere between 0.37 (IC_50_ for N617D) and 3.2 µM (2-fold IC_50_ for wt). The hampered Ca^2+^ selectivity and conductance mechanism of mutant DHPR(E292Q) (Figure 5—figure supplement 1a) is expected to be essentially the same as discussed above for DHPR(E1014K). In brief, low-affinity Ca^2+^ binding to the QEEE locus (Figure 5—figure supplement 1a) cannot support the crucial Ca^2+^ - Ca^2+^ repulsion mechanism and thus, Ca^2+^ conductance through mutant DHPR(E292Q) is blocked. Likewise, Ca^2+^ block of the bidirectional flux of monovalent cations, and hence Ca^2+^ selectivity is abolished.

Lastly, a third evolutionary concept also yielding a Ca^2+^ non-conducting DHPR was identified in the fast skeletal muscle of teleost fishes (Schredelseker et al., 2010). Although in the early phylogenetic teleost species (including zebrafish from the order *cypriniformes*) mutation N→D (N617D, mouse numbering) is the archetypical mutation to block DHPR Ca^2+^ influx, in phylogenetically higher developed teleost species starting with the order *lophiiformes* (anglerfishes), this negatively charged D was lost by mutating to a neutral T (Schredelseker et al., 2010). Concurrent to this D→T mutation, DHPR Ca^2+^ non-conductivity was re-installed by mutation of another D, which is one of the negative charges in the DCS locus (located in pore repeat I) and highly homologous in all mammalian L-type Ca^2+^ channels, to positively charged K (D→K). As demonstrated previously (Schredelseker et al., 2010), exchange of this DCS locus D_296_ with K in a GFP-tagged rabbit DHPRα_1S_ clone yielded mutant DHPR(D296K). Upon heterologous expression in *dysgenic* myotubes, this single charge conversion was sufficient to abolish inward Ca^2+^ currents. According to our combined model of Ca^2+^ selectivity and conductance and illustrated in Figure 5—figure supplement 1b, K_296_ does not permit formation of an active DCS locus, and thus Ca^2+^ from the t-tubular (extracellular) space is no more attracted to the DCS locus. Resultantly, there is lack of easy to mobilize low-affine DCS-bound Ca^2+^ that would compete with the tightly EEEE-bound Ca^2+^ for the binding valences of the EEEE locus (Figure 5—figure supplement 1b). Thus, the Ca^2+^- Ca^2+^ repulsion mechanism (Sather and McCleskey, 2003) and pushing out of the Ca^2+^ bound to the selectivity filter into the cytosol cannot take place. The surprising implication of charge conversion D296K in blocking of inward DHPR Ca^2+^ flux proves the importance of the DCS locus for proper inward DHPR Ca^2+^ currents in skeletal muscle and consequently, fundamentally supports our model of DHPR Ca^2+^ selectivity and Ca^2+^ conductivity.

## Materials and methods

**Key resources table:** 

Reagent type (species) or resource	Designation	Source or reference	Identifiers	Additional information
Strain, strain background (*Mus musculus*)	*nc*DHPR	doi:10.1038/s41467-017-00629-x Dayal et al., 2017		
Chemical compound, drug	(±)Bay K 8644	Sigma-Aldrich	Cat#: B112	10 µM
Chemical compound, drug	Nifedipine	Sigma-Aldrich	Cat#: N7634	10 µM
Chemical compound, drug	Tetraethylammonium chloride (TEA-Cl)	Sigma-Aldrich	Cat#: T2265	145 mM
Chemical compound, drug	N-benzyl-p-toluene sulphonamide (BTS)	Santa Cruz Biotechnology, Inc	Cat#: sc-202087	100 µM
Software, algorithm	MaxChelator simulation program	https://somapp.ucdmc.ucdavis.edu/pharmacology/bers/maxchelator/	RRID:SCR_018807	
Software, algorithm	ClampFit	Axon Instruments		version 10.7
Software, algorithm	SigmaPlot	Systat Software, Inc.	RRID:SCR_010285	version 11.0
Software, algorithm	GraphPad Prism	GraphPad Software, LLC	RRID:SCR_002798	version 8
Software, algorithm	PEP-FOLD 3.5	RPBS web portal		Version 3.5
Software, algorithm	GROMACS	University of Stockholm, University of Upsala	RRID:SCR_014565	version 2019.2
Software, algorithm	MOE	Chemical Computing Group ULC	RRID:SCR_014882	version 2020.01
Software, algorithm	AMBER	University of California, San Francisco.	RRID:SCR_014230	Version 2020
Software, algorithm	PyMOL	Schrödinger, LLC	RRID:SCR_000305	Version 2.4.0

### Animals

Generation of the Ca^2+^ non-conducting (*nc*)DHPR knock-in mouse strain, carrying a point mutation in the *Cacna1s* gene coding for N617D in pore loop II was described previously (Dayal et al., 2017). Animal breeding, care and maintenance was conducted in compliance with the guidelines of the EU Directive 2010/63/EU and approved by the Austrian Ministry of Science (BMWF-5.031/0001-II/3b/2012). Mice were housed in a controlled environment with a 12/12 hr light/dark cycle and had access to food and water ad libitum.

### Isolation and culture of skeletal myotubes

Primary myoblasts from new born up to 4-day-old pups homozygous for the non-conducting L-type Ca^2+^ channel mutant DHPR(N617D) or wild-type channel were enzymatically isolated and cultured in a humidified 37°C incubator with 5% CO_2_ as described previously (Dayal et al., 2017). Myotubes were maintained in growth medium consisting of Dulbecco’s modified Eagle’s medium supplemented with 10% fetal calf serum, 10% horse serum, 25 mM HEPES, 4 mM L-glutamine, and 1x penicillin/streptomycin and later replaced with differentiation medium (no fetal calf serum and only 2% horse serum).

### Whole cell patch clamp

Ionic currents were evoked by a standard 200 ms voltage-step protocol from −50 to +80 mV in 10 mV increments from a holding potential of −80 mV (Dayal et al., 2017), unless otherwise stated. To reduce inward currents via endogenous Na_V_ and T-type Ca^2+^ channels, every test pulse was preceded by a 1 s prepulse to −30 mV followed by a 50 ms repolarization to −50 mV (Adams et al., 1990). Borosilicate glass patch pipettes had resistance of 2–3 MΩ when filled with (in mM) 145 Cs-aspartate, 2 MgCl_2_, 10 HEPES, 0.1 Cs_2_-EGTA, and 2 Mg-ATP (pH 7.4 with CsOH). The standard bath solution for recording Ca^2+^ currents contained (in mM): 10 CaCl_2_, 145 TEA-Cl and 10 HEPES (pH 7.4 with TEA-OH). Myosin-II blocker BTS (100 µM, Sigma) was constantly present in the bath solution.

To test if depolarization-induced potentiation protocols known to promote mode 2 gating in L-type Ca^2+^ channels could evoke currents through DHPR(N617D), strong or long depolarizations in the presence of racemic 1,4-dihydropyridine (DHP) agonist (±)Bay K 8644 (10 µM) were performed (Bannister and Beam, 2011; Bannister and Beam, 2013). Pulse protocol for strong depolarization is depicted in Figure 1b. Briefly, 200 ms depolarization to either +90 mV or +60 mV is followed by a +60 mV pulse for 100 ms and finally by a repolarization to −20 mV for 70 ms. For long depolarization (Figure 1c), prolonged 2 s pulses from +10 - +80 mV in 10 mV increments were applied starting from a holding potential of −80 mV with an intermediate repolarizing step to −50 mV.

To investigate if DHPR(N617D) conducts slow-activating, non-inactivation inward Na^+^ currents, 145 mM TEA-Cl in standard bath solution was replaced by 145 mM NaCl (pH 7.4 with NaOH) to achieve near physiological Na^+^ concentration (150 mM). Furthermore, to test if these Na^+^ currents were also subject to block by Ca^2+^, 10 mM Ca^2+^ was reduced to near physiological 1 mM Ca^2+^.

To assess Ca^2+^ pore-binding affinity, dose-inhibition experiments for Ca^2+^ block of inward Li^+^ currents were performed. The bath solution for recording Li^+^ currents contained (in mM): 100 LiCl, 10 HEPES, 10 EGTA, and 25 for CaCl_2_ plus TEA-Cl (pH 7.4 with TEA-OH). Desired free Ca^2+^ concentrations (0 to 30 µM) were obtained by calibrating CaCl_2_ and TEA-Cl concentrations calculated using the MaxChelator simulation program (https://somapp.ucdmc.ucdavis.edu/pharmacology/bers/maxchelator/) (Supplementary File Table 1).

To test if the inward Li^+^ currents under external free [Ca^2+^]=0 as well as the slow outward and fast inward currents recorded under external 150 mM Na^+^ and 1 mM Ca^2+^ are mediated by DHPR(N617D) in *nc*DHPR myotubes, 10 µM of the 1,4-DHP antagonist nifedipine was added to the respective bath solutions.

All recordings were performed at room temperature using the Axopatch 200B amplifier (Axon Instruments Inc, CA), filtered at 1 kHz and sampled at 5 kHz.

### Data and statistical analysis

Data were analyzed and plotted using ClampFit (v10.7; Axon Instruments), SigmaPlot (v11.0; Systat Software, Inc) and Prism 8 (GraphPad Software, LLC). Data are represented as mean ± SEM and *n* = number of myotubes. Statistical significance was calculated using unpaired Student’s *t*-test, unless otherwise stated and was set as follows: *p<0.05, **p<0.01, and ***p<0.001.

### Structure preparation and molecular dynamics simulations

Atomic models were based on the cryo-EM structure of the rabbit DHPRα_1S_ - verapamil complex with a dilated intracellular gate associated to the binding of the phenylalkylamine Ca^2+^ antagonist drug verapamil (PDB accession number 6JPA) (Zhao et al., 2019). The structure of mutant DHPR(N617D) was derived from wt DHPR structure by replacing N_617_ with the negatively charged residue D_617_ and carrying out a local energy minimization using MOE (Molecular Operating Environment, Chemical Computing Group, version 2020.01). For simulations, we removed the voltage-sensing domains and truncated the S5 and S6 helices of each repeat, keeping the last nine residues of the S5 and S6 helices. The C- and N-termini of each repeat were capped with acetylamide (ACE) and N-methylamide to avoid perturbations by free charged functional groups. The starting structures for simulations were prepared in MOE using the Protonate3D tool (Labute, 2009). To neutralize the charges, we used the uniform background charge (Case et al., 2020; Hub et al., 2014). Using the tleap tool of the AmberTools20 package (Case et al., 2020; Roe and Cheatham, 2013), crystal structures were soaked in cubic water boxes of TIP3P water molecules with a minimum wall distance of 10 Å to the protein (Jorgensen et al., 1983; El Hage et al., 2018; Gapsys and de Groot, 2019). We added a total of 10 Ca^2+^ ions, corresponding to a concentration of approximately 10 nM. For all simulations, parameters of the AMBER force field 14 SB were used (Maier et al., 2015). The structures were carefully equilibrated using a multistep equilibration protocol (Wallnoefer et al., 2011).

For both wt DHPR and mutant DHPR(N617D), 10 ns of molecular dynamics (MD) simulations were performed in an isothermal - isobaric (NpT) ensemble using the GPU MD simulation engine pmemd.cuda (Salomon-Ferrer et al., 2013) to further equilibrate the structures in the presence of the Ca^2+^ ions. Bonds involving hydrogen atoms were restrained by applying the SHAKE algorithm (Miyamoto and Kollman, 1992), allowing a time step of 2 fs. Atmospheric pressure of the system was preserved by weak coupling to an external bath using the Berendsen algorithm (Berendsen et al., 1984). The Langevin thermostat (Doll et al., 1975; Adelman, 1976) was used to maintain the temperature at 300 K during simulations.

### Metadynamics simulations

Metadynamics is a powerful method to explore the properties of multidimensional free energy landscapes and to enhance the sampling of configurational space in reasonable computing time (Barducci et al., 2011). Metadynamics reconstructs the free energy surface as a function of few selected degrees of freedom, referred to as collective variables (CV), which accelerate rare events in the systems. The CVs should be able to characterize the key features of physical behavior of interest, distinguish between all different metastable states, and include the slow degrees of freedom. In metadynamics, an external history-dependent repulsive bias potential function constructed as a sum of Gaussians is deposited along the trajectory in the CV space and thereby, discourages revisiting and oversampling of same configurations. For metadynamics simulations, we used the GROMACS version 2019.2. The aim of the metadynamics simulation was to capture the movement of Ca^2+^ ions along the selectivity-filter conducting pathway and their passing through the EEEE motif. As CV, we chose the distance between the center of masses (COM) of the EEEE motif residues and the upper Ca^2+^ ion.

Simulations were performed at 300 K in an NpT ensemble. We used a Gaussian height of 1.5 kJ/mol and width of 0.1 nm. For both wt DHPR and mutant DHPR(N617D), five repetitions of metadynamics runs, each 10 ns, were performed. Pymol Molecular Graphics System was used to visualize the key interactions and differences between wt DHPR and mutant DHPR(N617D) pore conductances.

## Data Availability

All data generated or analyzed during this study are included in the manuscript and supporting files.

## Additional files

Supplementary file 1. Composition of external solutions with different free Ca^2+^concentrations used for recording Ca^2+ ^block of inward I_Li+_. The indicated free Ca^2+^ concentrations in the bath solutions were achieved by adjusting the concentrations of CaCl_2_ and TEA-Cl, calculated using the MaxChelator simulation program (https://somapp.ucdmc.ucdavis.edu/pharmacology/bers/maxchelator/).

Transparent reporting form
