# Supplementary material for: Pore mutation N617D in the skeletal muscle DHPR blocks Ca2+ influx due to atypical high-affinity Ca2+ binding
Source: eLife. 2021 Jun 1;10:e63435. doi: 10.7554/eLife.63435 (PMC8184209; doi:10.7554/eLife.63435)
Supplement: Supplementary file 1. — The indicated free Ca2+ concentrations in the bath solutions were achieved by adjusting the concentrations of CaCl2 and TEA-Cl, calculated using the MaxChelator simulation program (https://somapp.ucdmc.ucdavis.edu/pharmacology/bers/maxchelator/). [file elife-63435-supp1.docx]

**Supplementary file Table 1**

| **Free [Ca^2+^]** | **LiCl** | **HEPES** | **EGTA** | **CaCl_2_** | **TEA-Cl** |
| --- | --- | --- | --- | --- | --- |
|  | **(mM)** | | | | |
| **0** | 100 | 10 | 10 | 0 | 25 |
| **10 nM** | 100 | 10 | 10 | 0.79 | 24.21 |
| **30 nM** | 100 | 10 | 10 | 2.04 | 22.96 |
| **100 nM** | 100 | 10 | 10 | 4.60 | 20.4 |
| **300 nM** | 100 | 10 | 10 | 7.19 | 17.81 |
| **1 µM** | 100 | 10 | 10 | 8.95 | 16.05 |
| **3 µM** | 100 | 10 | 10 | 9.63 | 15.37 |
| **10 µM** | 100 | 10 | 10 | 9.89 | 15.11 |
| **30 µM** | 100 | 10 | 10 | 10 | 15 |
